# Phase I/II study of daily carboplatin, 5-fluorouracil and concurrent radiation therapy for locally advanced non-small-cell lung cancer

**DOI:** 10.1038/sj.bjc.6601227

**Published:** 2003-08-26

**Authors:** H Yoshizawa, J Tanaka, H Kagamu, Y Maruyama, H Miyao, K Ito, T Sato, A Iwashima, E Suzuki, F Gejyo

**Affiliations:** 1 Niigata Lung Cancer Treatment Group, Niigata University Graduate School of Medical and Dental Sciences, Niigata 951-8510, Japan; 2 Division of Respiratory Medicine, Niigata University Graduate School of Medical and Dental Sciences, Niigata 951-8510, Japan

**Keywords:** carboplatin, 5- fluorouracil, thoracic radiation therapy

## Abstract

A study was undertaken to determine the maximum tolerated dose, the dose-limiting toxicities and the response rate of carboplatin and 5-fluorouracil administered daily with concurrent thoracic radiation therapy in patients with locally advanced non-small-cell lung cancer. In a phase I/II clinical trial, patients with histologically documented, unresectable stage IIIA or IIIB non-small-cell lung cancer (NSCLC) were enrolled. Carboplatin (20–40 mg m^−2^ 2-h infusion, daily) and 5-fluorouracil (200 mg m^−2^ 24-h continuous infusion, daily) were administered concurrently with radiotherapy on days 1–33. Radiotherapy, with a total dose of 60 Gy, was delivered in 30 fractions on days 1–40. In the phase I portion, the daily dose of carboplatin was escalated from 20 to 40 mg m^−2^. Once the maximum tolerated dose (MTD) and recommended dose (RD) of carboplatin was determined, the study entered the phase II portion. In the phase I portion, the daily MTD and RD of carboplatin were 40 and 35 mg m^−2^, respectively. The dose-limiting toxicity was neutropenia. In the following phase II study, 30 patients were entered and the objective response rate was 76.7% (95% CI, 62–92%) and the local control rate was 85.7%. The median survival time was 19.8 months, with a survival rate of 70% at 1 year, 36.7% at 2 years. The major toxicities of treatment were neutropenia (⩾grade 3, 87.9%) and thrombocytopenia (⩾grade 3, 23.3%). This combined therapy for locally advanced non-small-cell lung cancer is promising and shows acceptable toxicity.

Non-small-cell lung cancer (NSCLC) accounts for approximately 75% of all lung cancers (Travis *et al*, 1995). Only a low percentage of patients present a disease susceptible to surgical resection. In fact, approximately 40% of patients with NSCLC present with locally or regionally advanced tumours that are ineffectively treated by primary surgery. Large studies have shown that a small percentage of those patients can be cured with radiotherapy and even after using the best available radiation schedules, recurrences in the radiation field are observed in a substantial percentage of the patients (Cox *et al*, 1990; Arriagada *et al*, 1991; Graham *et al*, 1995).

Due to the limited benefits provided by radiation therapy alone, the use of combined chemoradiotherapy in the patients has been explored. A combination of chemotherapy and radiotherapy has been shown to improve the outcome of the patients with locally advanced NSCLC, achieving a median survival of 13–14 months and 5-year survival rates of 13–20% (Dillman *et al*, 1990,Dillman *et al*, 1996; Schaake-Koning *et al*, 1992; Sause *et al*, 1995).

In the EORTC phase III study, 08844 Schaake-koning *et al* (1992) demonstrated that radiotherapy combined with daily administration of low-dose cisplatin resulted in improved local control and actuarial survival in patients with unresectable NSCLC. In that study, daily administration of cisplatin proved to be more effective than a weekly schedule in potentiating the local tumour control achievable with radiation alone. In addition, Jeremic *et al* (1996) reported that the combination of radiation therapy and low-dose daily carboplatin (CBDCA) plus etoposide improved the survival of patients with stage III NSCLC as a result of improved local control. These results suggest that concurrent low-dose daily administration of platinum compounds enhance local control by radiation therapy, resulting in better survival of stage III NSCLC.

Many agents have been reported to have a radiosensitising effect. 5-fluorouracil (5-FU) was demonstrated to be a radiosensitiser in a preclinical study (Byfield *et al*, 1982). A randomised study using single-agent 5-FU in conjunction with radiotherapy suggested that, in terms of local control and patient survival, it improved the effect of radiotherapy alone (Lo *et al*, 1976). Although inhibition of repair of radiation-induced DNA damage has been postulated to occur, the precise cellular mechanisms by which 5-FU and radiotherapy interact have not been defined (Schilsky, 1992).

In addition to the raidosensitising effect of these agents, a combination of platinum compound and 5-FU has been shown to have synergistic antitumour activity in both preclinical and clinical studies (Scanlon *et al*, 1986; Olver *et al*, 1989; Esaki *et al*, 1992; Saikawa *et al*, 1994). The mechanism of synergism of these two agents remains unclear, although there are various hypotheses concerning the modulatory effect of platinum compounds on 5-FU. It was suggested that the platinum-induced intracellular folate level potentiates the effect of 5-fluorodeoxyuridine monophosphate by forming a covalent ternary complex with thymidylate synthase, leading to enhanced 5-FU cytotoxicity (Scanlon *et al*, 1986).

These results prompted us to conduct a phase I–II trial of carboplatin and continuous infusion of 5-FU in combination with concurrent radiotherapy in patients with locally advanced NSCLC. The objective of the trial was to determine the maximum tolerated dose of carboplatin that could be used in combination with continuous infusion of 5-FU and concurrent radiotherapy. The efficacy and toxicity of the combination were further evaluated in the following phase II study.

## MATERIALS AND METHODS

### Patient eligibility

Previously untreated patients with histologically documented inoperable stage IIIA or stage IIIB NSCLC were eligible for this study. Patients with a malignant or exudative pleural effusion were not eligible. All patients had measurable or assessable disease. Further eligibility criteria included: age greater than 20 and less than 75 years; ECOG performance status ⩽2; no prior chemotherapy; no prior lung radiation therapy; platelet count ⩾100 000 *μ*l^−1^; white blood cell (WBC) count 3000 *μ*l^−1^; haemoglobin >10 g dl^−1^; BUN <1.5-fold the upper limit of normal (ULN); creatinine level <1.1 mg dl^−1^; bilirubin <1.5-fold the upper limit of normal (ULN); AST <two-fold the ULN; 24-h creatinine clearance ⩾60 ml min^−1^; PaO_2_>65 mmHg in a sample of arterial blood; and no other serious medical or psychiatric illness. Height, weight, performance status and tumour stage were recorded. Before initiation of protocol therapy, all patients underwent a history and physical examination; complete blood cell count (CBC) with differential and platelet count; chemistry panel; posterior–anterior (PA) and lateral chest radiography; computed tomographic (CT) scan of the head, chest, and upper abdomen (to include the liver and adrenals); and a bone scan. The protocol was approved by the Institutional Ethics Board. All patients gave written informed consent.

### Chemotherapy

**Figure 1:**
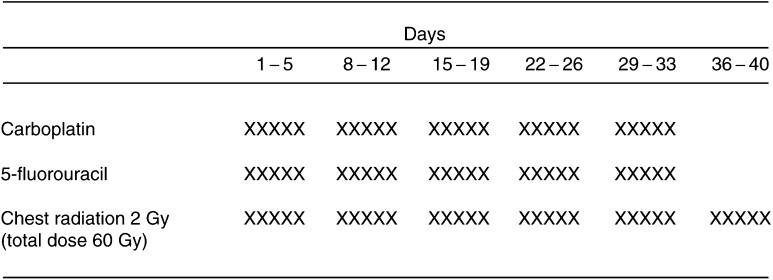
Treatment schema for daily carboplatin, 5-FU and concurrent radiation therapy.

Carboplatin (20–40 mg m^−2^ 2-h infusion), and 5-FU (200 mg m^−2^ 24-h continuous infusion) were administered concurrently with radiotherapy on days 1–33 (Figure 1

). Carboplatin was administered as a 2-h intravenous infusion 1–2 h before irradiation. The daily dose of carboplatin was increased stepwise from 20 to 40 mg m^−2^ in 5 mg increments. 5-FU (200 mg m^−2^) was administerd as a 24-h continuous infusion. When grade 3 or higher haematological toxicity occurred, chemotherapy was withheld until toxicity recovered to ⩽grade 2.

### Radiation therapy

Chest irradiation was started concurrently on day 1 for all patients using a linear accelerator (6–10 MeV). A total dose of 60 Gy was delivered in 30 fractions at five fractions per week over 6 weeks. The treatment volumes consisted of original and boost volumes. The initial dose of 40 Gy was administered to the original volume, which included the site of the primary tumour with a margin of 2 cm around the mass and the ipsilateral hilum, and the whole width of the mediastinum with a margin of 1 cm around the radiographically demonstrated tumour mass. The supraclavicular region was further included if involved with the tumour. An additional 20 Gy was administered to the boost volume that included the site of the primary tumour and involved lymph nodes. The original volume was treated with an anterior–posterior parallel–opposed pair of portals, and the boost volume was treated with the same pair or with a pair of oblique fields if the cumulative radiation dose to the spinal cord was greater than 45 Gy.

When grade 3 or higher radiation esophagitis toxicity or grade 4 haematological toxicity occurred, radiation therapy was withheld until those toxicities recovered to ⩽grade 2.

### Toxicity and response evaluation

Toxicity was graded using the WHO scale for acute and subacute toxicity. The radiation-induced effect on normal tissue was assessed as either acute or late toxicity, according to the RTOG/EORTC criteria (Cox *et al*, 1995). A minimum of three patients were evaluated per dose level. The maximum-tolerated dose (MTD) was defined as the dose level that produced grade 4 myelosuppression (⩾4 days) and grade 3–4 nonhaematologic toxicity (excluding alopecia) in two-thirds of the patients. If none of the three patients experienced dose-limiting toxicity (DLT), the subsequent patients were treated at the next dose level. If one of the three patients experienced DLT, more patients were entered at the same dose level. Complete blood cell count measurement was performed at least twice weekly, while routine chemistry, urine analysis and chest X-rays were performed once per week during chemotherapy.

The standard response criteria used were as follows: complete response (CR) was defined as the disappearance of all measurable disease and complete disappearance of all signs, symptoms and biochemical evidence of tumour activity for at least 4 weeks; partial response (PR) was defined as a 50% or greater decrease in the product of the perpendicular measurements of disease with no progression in any lesion and no new lesions identified; no change (NC) was defined as no evidence of progressive disease or any measurable response less than a PR; progressive disease (PD) was defined as a greater than 25% increase in the product of the longest perpendicular diameters of any measurable lesion or the appearance of new lesions.

### Statistical analysis

The phase II part of this study was designed to detect a response rate of 75% compared with a minimal, clinically meaningful response rate of 50%. A sample size of 30 patients was required with an *α* error of 0.1 and *β* error of 0.1. Survival analysis was performed using the Kaplan–Meier method (Fleming, 1982).

## RESULTS

### Patient characteristics

The patient characteristics are listed in Table 1

**Table 1:** Patient characteristics

**Characteristic**	**No. of patients(%)**
No. of patients entered	46(
No. of patients eligible	46(
Gender	
Male	42 (91.3)
Female	4 (8.7)
ECOG performance status	
0	12 (26.1)
1	34 (73.9)
2	0
Histology	
Squamous cell carcinoma	24 (52.2)
Adenocarcinoma	22 (47.8)
Stage of disease	
IIIA	26 (56.5)
IIIB	20 (43.5)

. Forty-six patients (26 with IIIA, 20 with IIIB) were entered into the study between February 1995 and March 1999. In all, 42 were male and four were female patients; the median age was 59.5 years (range, 39–71). The ECOG PS was as follows: 12 patients of PS 0, 34 patients of PS 1.

In total, 22 patients were treated in the phase I portion of the study: three patients at the carboplatin dose level of 20 mg m^−2^ day^−1^, three patients at 25 mg m^−2^ day^−1^, four at 30 mg m^−2^ day^−1^, six at 35 mg m^−2^ day^−1^ and six at 40 mg m^−2^ day^−1^. After the determination of the MTD and recommended dose (RD) of daily carboplatin, 24 patients were entered into the phase II part of the trial.

### Toxicity and MTD determination

Toxicity was evaluated for all eligible patients. The recommended dose of daily carboplatin was deemed to be 35 mg m^−2^ because at 40 mg m^−2^, four of the six patients experienced DLT as defined by the protocol; grade 4 neutropenia persisted for over 4 days (Table 2

**Table 2:** Haematological and nonhaematological toxicity in the phase I study

		**Neutropenia**		**Thrombocytopenia**		**Oesophagitis**		**Pneumonitis**	
**Carboplatin dose level (mg m^−2^)**	**No. of patients**	**Grade 3**	**Grade 4**	**Grade 3**	**Grade 4**	**Grade 2**	**Grade 3**	**Grade 2**	**Grade 3**
20	3	1	0	0	0	0	0	0	0
25	3	2	0	1	0	1	0	0	0
30	4	2	0	1	0	0	0	0	0
35	6	4	2	4	0	0	0	0	0
40	6	2	4	4	0	2	0	0	0

). Grade 3 thrombocytopenia was frequently observed at level 5 (carboplatin dose 40 mg m^−2^) and three cases required to infuse platelet concentrates. Grades 1–2 oesophagitis was observed at all dose levels. In a subsequent phase II study, 30 patients including six patients treated at the RD in the phase I study were entered. Haematological and nonhaematological toxicities of the study are listed in Table 3

**Table 3:** Haematological and nonhaematological toxicity in the phase II study

	**No. of patients with toxicities**				
**Toxicity Grade**	**1**	**2**	**3**	**4**	**Grade 3–4(%)**
Neutropenia	0	3	22	3	87.9
Thrombocytopenia	10	9	4	3	23.3
Anaemia	14	7	0	0	0
Nausea/vomiting	9	0	0	0	0
Diarrhoea	6	0	0	0	0
Fever	3	1	0	0	0
Peripheral neurotoxicity	5	0	0	0	0
Oesophagitis	19	6	1	0	3.3
Pneumonitis	20	5	2	0	6.7

. Neutropenia was the most severe toxicity noted in this study. Three patients (10%) had grade 4 neutropenia which persisted for 3–5 days (mean 4 days), and both chemotherapy and radiation therapy were interrupted for 4–7 days (mean 5.7 days). These patients underwent rhG-CSF administration. Among them, one patient experienced grade 2 fever and all the patients experienced grade 1–2 oesophagitis, except one case who had grade 3 at a final week of radiation therapy. Two patients had grade 3 acute pulmonary toxicity and required a transient oxygen supply. Due to this acute toxic effect, irradation was interrupted for 7 and 16 days. Grade 2 late toxicity (RTOG) was observed in two patients who had symptomatic radiation pulmonary fibrosis.

### Response and survival

Of the 30 evaluable patients in the phase II part of the study, two patients (6.7%) achieved a CR and 21 (70%) achieved a PR for an overall response rate of 76.7% (95% CI, 62–92%). Local control was achieved in 85.7% of the patients and distant metastasis was the reason for treatment failure of all three cases evaluated as PD.

**Figure 2:**
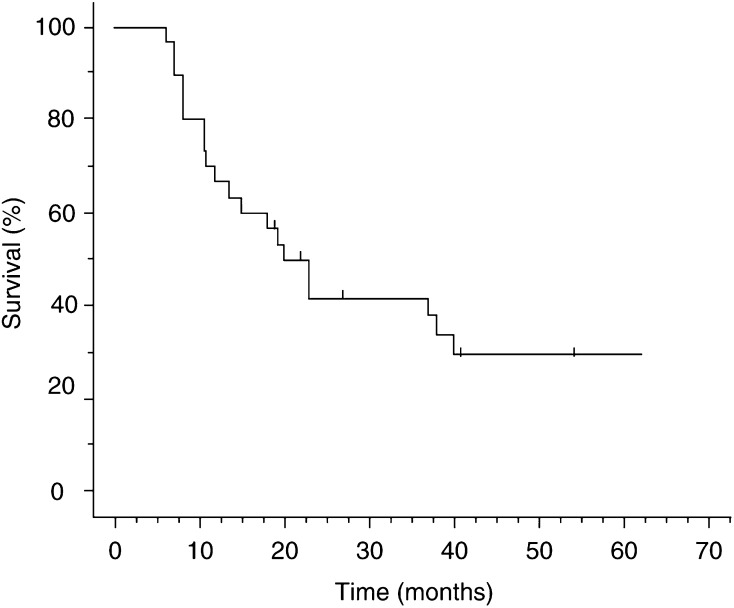
Survival rate–the 1-year survival rate was 70%.

The 1-year overall survival rate was 70% and the 2-year survival rate was 36.7% (Figure 2

). The median overall survival time and median follow-up time was 19.8, and 25.8 months, respectively. The median progression-free survival time was 11.1 months.

### Pattern of failure

In all, 15 of 23 responding patients had relapsed. The median time to progression of responding patients was 13.9 months. Distant metastases alone were the primary site of disease progression in seven (47%) patients, three (20%) developed locoregional only progression and five (33%) developed both distant metastases and locoregional progression. Of the patients with distant metastases, the brain was the most common site of failure with three (25%) patients presenting with brain-only metastases, three (33%) with brain and bone metastases and two (17%) with brain and adrenal gland metastases. Two patients developed metastases in the adrenal gland alone and the remaining two patients presented with liver metastases alone.

## DISCUSSION

Approximately 40% of patients with newly diagnosed NSCLC initially present with locally advanced disease, and the majority are inoperable. Thoracic radiation therapy (TRT) provides local control and effective palliation of symptoms, but has minimal effect on survival for this set of patients. The addition of chemotherapy to TRT sequentially (Dillman *et al*, 1990; Le Chevalier *et al*, 1992; Sause *et al*, 1995) or concurrently (Jeremic *et al*, 1995,Jeremic *et al*, 1996; Schaake-Koning *et al*, 1992) has shown an impact on survival among patients with unresectable NSCLC, especially those with good performance status and <5% weight loss (Sause *et al*, 1995), compared with TRT alone. Recent progress in the management of locally advanced NSCLC came about in part because of the recognition that cisplatin-based chemotherapy imparts a modest but real survival benefit in this set of patients. With a newer combination of chemotherapy regimens containing vinorelbin, gemcitabine or paclitaxel, additional albeit modest survival gains have been realised in recent years. Thus, the survival of patients with locally advanced, unresectable NSCLC has improved in part because of more active chemotherapy regimens.

However, improving local tumour control is a key factor for optimising survival. Efforts to improve local tumour control are apparently worthwhile because enhanced local control can translate into improved survival even in the absence of improved control of extrathoracic metastases (Perez *et al*, 1986; Schaake-Koning *et al*, 1992; Jeremic *et al*, 1996; Saunders *et al*, 1999). The strategies designed to improve local control include (a) the use of radiation-sensitising drugs, (b) the use of fractionation of radiations and (c) escalated radiation doses with three-dimensional (3-D) treatment planning.

Regarding radiation-sensitising drugs, several common antineoplastic agents such as cisplatin, etoposide, paclitaxel and gemcitabine among others have the potential to act as radiation-sensitising agents. Although newer drug regimens combined with radiotherapy can be expected to improve the clinical outcome further, increased host toxicities may cause the interruption of the treatment or dose reduction of one or both treatment modalities (Yokoyama *et al*, 1998; Blackstock *et al*, 2001).

We designed this phase I/II study in an attempt to improve local tumour control, which had been shown to affect the survival of patients with stage III NSCLC. Both carboplatin and 5-FU selected for the current study were demonstrated to be radiosensitisers in preclinical (Byfield *et al*, 1982; Begg *et al*, 1987; Havemann *et al*, 1992) and clinical studies (Lo *et al*, 1976; Wolf *et al*, 1992; Belani and Aisner, 1994; Belani *et al*, 1996). In addition, a combination of platinum compound and 5-FU was shown to have synergistic antitumour activity (Scanlon *et al*, 1986; Olver *et al*, 1989; Esaki *et al*, 1992; Saikawa *et al*, 1994). In the phase I part of the current study, the recommended dose of daily carboplatin was determined to be 35 mg m^−2^ because at 40 mg m^−2^, patients experienced grade 4 neutropenia and/or grade 3 thrombocytopenia. The dose of 5-FU (200 mg m^−2^) was determined according to the previous trials that were aimed to improve local control by radiation therapy (Hansen *et al*, 1987; Ngan *et al*, 2001; Nishimura *et al*, 2002).

In contrast to the haematological toxicities, severe radiation oesophagitis was not observed at any dose levels. In a subsequent phase II study, two patients (6.7%) achieved a CR and 21 (70%) achieved a PR for an overall response rate of 76.7%. Local control was achieved in 85.7% of patients, and distant metastasis was the reason for treatment failure in all three cases evaluated as PD. In addition, the primary tumour of two cases evaluated as no change continued to reduce their size for several months and these cases were re-evaluated as PR. Including these cases, local control was achieved in 93.3% patients, which is favourable compared with previous findings.

Although the current study focused on the local control, the distinct goal of locally advanced NSCLC is preventing distant failure. In the trial of sequential full-dose chemotherapy by Le Chevalier *et al* (1994), the improved survival seen in the combined-modality arm was related entirely to a decrease in distant failure. In addition, the Southwest Oncology Group trial 9504 evaluated docetaxel as a consolidation therapy following concurrent chemoradiotherapy in stage III NSCLC. The results in 83 patients showed prolonged survival (median survival 26 months, 1-year survival 76% and 2-year survival 53%), the best results reported to date (Gandara *et al*, 2000). Based on the present results, a phase II trial that evaluates the feasibility and the efficacy of consolidation therapy following the current chemoradiotherapy is ongoing in our study group.

In conclusion, a combination of daily carboplatin and 5-FU with concurrent thoracic radiotherapy was found to be effective and feasible for the treatment of locally advanced NSCLC. Although the response rate and median survival were favourable compared with previously published phase II studies, additional consolidation chemotherapy to control distant micrometastasis needs to be considered to improve the clinical outcome further.
